# Ablation of human telomerase reverse transcriptase (hTERT) induces cellular senescence in gastric cancer through a galectin-3 dependent mechanism

**DOI:** 10.18632/oncotarget.10986

**Published:** 2016-08-01

**Authors:** Sun-Hyuk La, Seok-Jun Kim, Hyeok-Gu Kang, Han-Woong Lee, Kyung-Hee Chun

**Affiliations:** ^1^ Department of Biochemistry and Molecular Biology, Yonsei University College of Medicine, Seodaemun-gu, Seoul 03722, Republic of Korea; ^2^ Department of Biochemistry, College of Life Science and Biotechnology, Yonsei University, Seodaemun-gu, Seoul 03722, Republic of Korea; ^3^ Brain Korea 21 PLUS Project for Medical Science, Yonsei University, Seodaemun-gu, Seoul 03722, Republic of Korea

**Keywords:** telomerase, hTERT, galectin-3, cellular senescence, gastric cancer

## Abstract

The human Telomerase Reverse Transcriptase (hTERT) gene encodes a rate-limiting catalytic subunit of telomerase that maintains genomic integrity. Suppression of hTERT expression could induce cellular senescence and is considered a potent approach for gastric cancer therapy. However, control of hTERT expression and function remains poorly understood in gastric cancer. In this study, we demonstrated that high expression levels of hTERT in malignant tissues are correlated with poor survival probability in gastric cancer patients. Knockdown of hTERT expression retarded cell proliferation and cellular senescence, which was confirmed by increased protein expression levels of p21^cip1^ and p27^kip1^, and decreased phosphorylation of Rb. In contrast, overexpression of hTERT increased cell proliferation and decreased cellular senescence. Remarkably, the down-regulation of hTERT expression was detected in *lgals3^−/−^* mouse embryo fibroblasts (MEFs). Knockdown of galectin-3 decreased the expression of hTERT in gastric cancer cells. Galectin-3 ablation-induced cellular senescence was rescued by concomitant overexpression of hTERT. hTERT ablation-induced cellular senescence and p21^cip1^ and p27^kip1^ expression was rescued by concomitant overexpression of galectin-3. The size of tumor burdens was increased in hTERT-overexpressed gastric cancer cells xenografted mice, whereas it was repressed by concomitant depletion of galectin-3. Additionally, we determined that the N-terminal domain of galectin-3 directly interacted with hTERT. The telomeric activity of hTERT was also decreased by galectin-3 ablation. Taken together, ablation of hTERT induces cellular senescence and inhibits the growth of gastric cancer cells, suggesting that it could be a potent target in gastric cancer therapy. We also propose that galectin-3 is an important regulator of hTERT expression and telomeric activity in gastric tumorigenesis.

## INTRODUCTION

Gastric cancer is one of the most common malignancies worldwide. Although there has been increased knowledge of the key molecular signaling pathways involved in gastric carcinogenesis, the molecular composition of gastric cancer has not been fully understood [1, 2]. Currently, surgery is the only treatment strategy to cure relatively early-staged gastric cancer; however, for patients in an advanced stage, the overall survival is poor (approximately 10 months for those who receive conventional chemotherapy). Chemotherapy is the most common therapy for advanced gastric cancer, but its efficacy is limited. Targeted therapy is a new therapeutic approach that might improve the survival of advanced gastric cancer patients [3]. Therefore, molecular analyses have been vigorously performed for the last decades, and results suggest that alterations in the structure and function of oncogenes and tumor suppressor genes, genetic instability, as well as the acquisition of cell immortality, could be relevant in the pathogenesis of gastric cancer. Telomerase activation is believed to be a crucial event in most immortal cells and cancer cells [4]. However, its clinicopathologic significance and the mechanisms regulating telomerase activity remain to be clarified in gastric cancer.

Eukaryotic chromosomes are capped with repetitive telomere sequences that protect the chromosome ends against exonucleases and ligases, thus preventing fusion, recombination, and degradation [5]. Telomere maintenance is often accompanied by an activated telomerase to protect genetically damaged DNA from normal cell senescence; however, telomerase activation through the induction of human Telomerase Reverse Transcriptase (hTERT) is an essential step in gastric cancer [4] and other cancers [6], suggesting studying the functional maintenance of telomerase is important in tumorigenesis with senescence [5]. Cellular senescence, a state of irreversible cell cycle arrest, is a part of the aging program and it involves multiple signaling cascades [7]. In general, senescence can be divided into replicative senescence and premature senescence. Replicative senescence has been described for all metabolically active cells that undergo a spontaneous decline in growth rate. Notably, ectopic expression of hTERT can prevent it. In cancer cells, premature senescence induced by oncogenes, named oncogene-induced senescence, and plays an important role in preventing the development of cancer. This suggests that induction of cellular senescence by targeting hTERT is a potential approach for cancer therapy. Moreover, most gastric cancers show genetic instability, either microsatellite instability or chromosomal instability, which is considered an early event in gastric carcinogenesis [8]. Based on clinical pathology, up-regulated expression and activity of hTERT is a critical factor in gastric carcinogenesis, however, the regulatory mechanisms underlying hTERT expression and function in cellular senescence remains poorly understood in gastric cancer.

Previously, we reported that galectin-3 regulates cellular senescence without oncogenic stress in gastric cancer [9]. We also determined, using DNA microarray analysis, that ablation of galectin-3 decreased the mRNA expression of hTERT [10]. We confirmed that the expression of senescence inducers such as p21^cip1^ and p27^kip1^, was down-regulated by telomerase; moreover, galectin-3 also down-regulated their expression. The role of galectin-3 and human telomerase subunits with hTERT in cellular senescence was confirmed by senescence-associated β-galactosidase (SA-β-gal) activity. Therefore, in this study, we determined the interaction between hTERT and galectin-3 to regulate cellular senescence in gastric cancer. We demonstrate that hTERT regulates cellular senescence and that galectin-3 regulates hTERT expression and activation in gastric cancer.

## RESULTS

### Analysis of hTERT expression and its related survival probability in malignant tissues of gastric cancer patients

**Figure 1:**
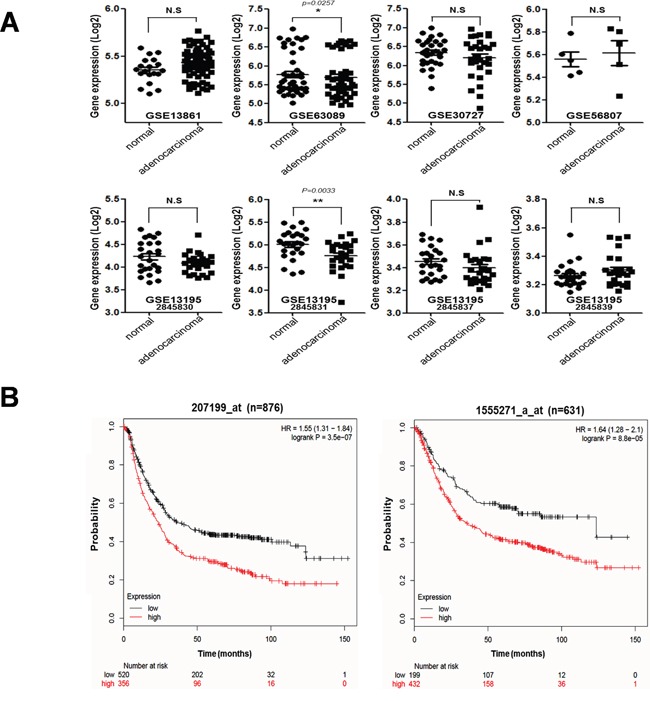
Correlation between tumorigenesis and hTERT expression in gastric cancer patients **A.** mRNA expression levels of hTERT of malignant tissues and normal tissues from gastric cancer patients are presented as a scatter diagram (GSE13861, GSE63089, GSE30727, GSE56807, GSE13195-2845830, 2845831, 2845837, 2845839). **B.** Kaplan–Meier survival plots demonstrating the poor prognostic effect due to the hTERT up-regulation, which correlated with a worse Overall Survival in gastric cancer patients (probe1:207199_at_n = 876, probe2:1555271_a_at_n = 631) (http://kmplot.com/analysis).

We determined the expression levels of hTERT in malignant stomach tissues from gastric cancer patients available in the NCBI database (Figure 1A) and performed a Kaplan–Meier analysis to generate a survival curve using the online resource http://kmplot.com/ and analyzed gene sets for gastric cancer patients (Figure 1B). We collected eight datasets of gastric cancer patients and compared the expression levels of hTERT in malignant and normal stomach tissues (Figure 1A). Among eight analyses, only two sets showed statistically increased expression of hTERT in malignant tissues, when compared to normal tissues, and six sets showed no statistical differences between malignant and normal tissues. However, statistical analysis showed that high expression of hTERT correlated with significant lower survival probability, when compared to low expression of hTERT in malignant stomach tissues from gastric cancer patients (Figure 1B).

### Knockdown of hTERT induces cellular senescence in gastric cancer cells

First, we determined expression levels of hTERT in twelve gastric cancer cell lines (Supplementary Figure 1). Among of them, YCC-2, SNU-16, SNU-216, and SNU719 cells expressed high mRNA levels of hTERT. Based on these results, we choose YCC-2 and SNU-216 cells for loss of function experiments, and MKN-28 and SNU-638 cells for gain of function experiments.

**Figure 2:**
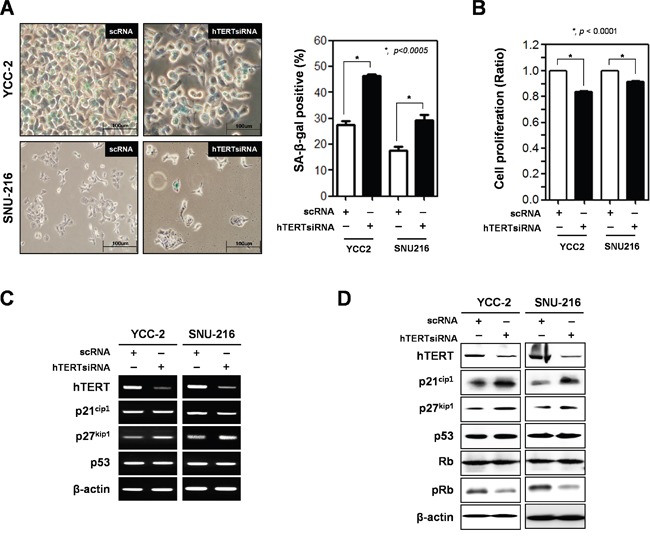
Ablation of hTERT altered the cell proliferation and cellular senescence of gastric cancer cells Both YCC-2 and SNU-216 gastric cancer cells were transfected with scrambled siRNA (scRNA) and hTERT specific siRNA for 48 h. **A.** Cellular senescence was detected by SA-β-galactosidase activity in YCC-2 and SNU-216 gastric cancer cells. The graph shows the percentage of SA-β-galactosidase-positive cells. (*P* < 0.0005) **B.** Cell proliferation was measured by WST assay in YCC-2 and SNU-216 gastric cancer cells. (*P* < 0.0001) **C.** Detection of mRNA expression levels of hTERT, p21^cip1^, p27^kip1^, and p53 were detected by RT-PCR analysis and **D.** protein expression levels of these were detected by western blot analysis in YCC-2 and SNU-216 gastric cancer cells after transfection with scrambled siRNA (scRNA) and hTERT specific siRNA. β-actin was used loading control.

After knockdown of hTERT by three kinds of 10 μM hTERT-specific siRNA for 2 days, determined the diminishment of hTERT expression in YCC-2 cells (Supplementary Figure 2). And then, we analyzed cellular senescence using the SA-β-gal assay (Figure 1A). Cellular senescence significantly increased in both cells lines (46.4% in YCC-2 cells and 27.6% in SNU-216 cells) compared to control cells. Cell proliferation was also reduced by knockdown of hTERT in both cell lines (Figure 2B). Therefore, we detected both mRNA and protein expression levels of senescence-related molecules (Figure 2A and 2B). Knockdown of hTERT slightly increased mRNA expression levels of p27^kip1^ (Figure 2C), whereas it drastically increased the protein expression levels of p21^cip1^ and p27^kip1^, known cell cycle inhibitors, but not p53. Phosphorylation of Rb was decreased by knockdown of hTERT (Figure 2D). We attempted to determine the expression levels of p16^INK4a^, but they could not be detected in these gastric cancer cells (data not shown).

### Overexpression of hTERT reduces cellular senescence in gastric cancer cells

**Figure 3:**
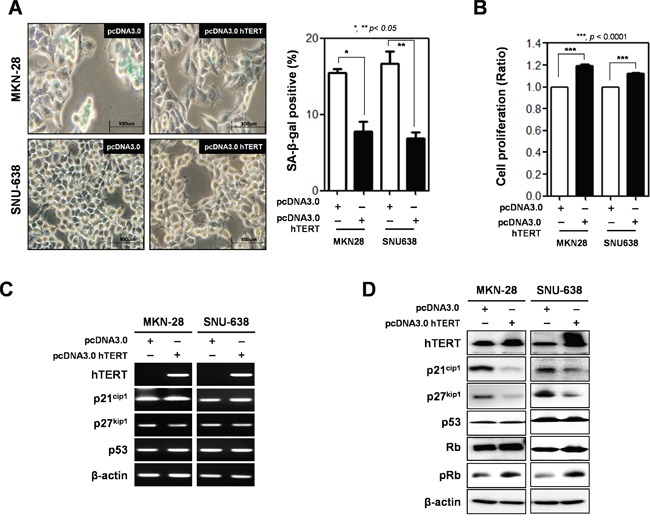
Overexpression of hTERT altered the cell proliferation and cellular senescence of gastric cancer cells Both MKN-28 and SNU-638 cells were transfected with pcDNA3.0 empty vector (pcDNA3.0) and pcDNA3.0_hTERT for 48 h. **A.** Cellular senescence was detected by β-galactosidase activity in MKN-28 and SNU-638 gastric cancer cells after transfection with a pcDNA3.0 empty vector (pcDNA3.0) or pcDNA3.0_hTERT. The graph shows the percentage of β-galactosidase-positive cells. **B.** Cell proliferation was examined in MKN-28 and SNU-638 gastric cancer cell lines after transfection with pcDNA3.0 and pcDNA3.0_hTERT (*P* < 0.0001). **C.** Detection of mRNA expression levels of hTERT, p21^cip1^, p27^kip1^, and p53 were detected by RT-PCR analysis and **D.** protein expression levels of these were detected by western blot analysis in MKN-28 and SNU-638 cells after transfection with pcDNA3.0 and pcDNA3.0 hTERT. β-actin was used loading control.

To determine whether hTERT overexpression reduced premature senescence, we prepared an hTERT overexpression vector (pcDNA3.0-hTERT) and transiently transfected it into SNU-638 and MKN-28 cells. Cellular senescence and cell proliferation were analyzed using the WST assay and SA-β-gal assay (Figure 3A and 3B). Overexpression of hTERT reduced cellular senescence in cells (15.4% to 7.75% in MKN-28 cells, and 16.7% to 6.9% in SNU-638 cells) (Figure 3A). Cell proliferation was increased in hTERT overexpressed SNU-638 cells and MKN-28 cells (Figure 3B). Both mRNA and protein expression levels of senescence-related molecules were also detected in hTERT overexpressed cells (Figure 3C and 3D). The mRNA expression levels of p21^cip1^, p27^kip1^, and p53 were not altered by hTERT overexpression (Figure 3C). Protein expression levels of p21^cip1^ and p27^kip1^ were significantly decreased, but not p53 expression levels. Phosphorylation of Rb was also increased by overexpression of hTERT (Figure 3D).

These results suggest that expression levels of hTERT are related with induction of cellular senescence in gastric cancer cells.

### Expression levels of hTERT are regulated by galectin-3 in gastric cancer cells

**Figure 4:**
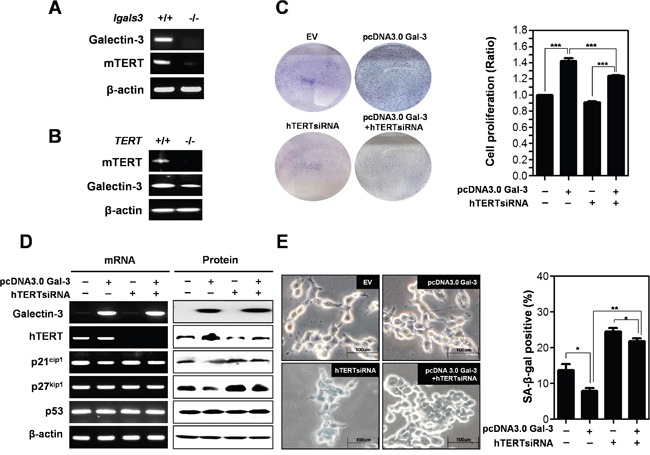
Detection of expression level of mTERT in galectin-3 knockout mouse embryo fibroblasts (*lgals3^−/−^* KO MEFs) and expression level of galectin-3 in mTERT knockout mouse embryo fibroblasts (*TERT* KO MEFs) **A.** mRNA expression levels of mTERT and Galectin-3 in *lgals3^−/−^* KO MEFs and **B.** mRNA expression levels of mTERT and galectin-3 in *TERT* KO MEFs were detected by RT-PCR analysis. β-actin was used loading control. **C-E.** SNU-638 gastric cancer cells were transfected with pcDNA3.0 alone, pcDNA3.0-galectin-3 alone, hTERT siRNA alone, and hTERT siRNA and pcDNA3.0-galectin-3 for 48 h. **C.** Each cell plate was stained with Crystal violet solution and showed by photographs (left panel), and cell proliferation in each cell plate was measured by WST assay and presented by quantitative graphs (right panel). **D.** Detection of mRNA expression levels of galectin-3, hTERT, p21^cip1^, p27^kip1^, and p53 were detected by RT-PCR analysis and protein expression levels of these were detected by western blot analysis in SNU-638 gastric cancer cells. β-actin was used loading control. **E.** Cellular senescence was detected by SA-β-galactosidase activity in SNU-638 cells. The graph shows the percentage of SA-β-galactosidase-positive cells.

Previously, we reported DNA microarray analysis results using galectin-3 depleted AGS gastric cancer cells [10]. We obtained data demonstrating that hTERT expression levels were also reduced in galectin-3 depleted cells (Supplementary Figure 3). Therefore, we determined mRNA expression levels and correlation between hTERT and galectin-3 in twelve gastric cancer cell lines (Supplementary Figure 1). Notably, we found that cell lines with high expression levels of hTERT also had high expression levels of galectin-3 (YCC-2, SNU-16, SNU-216, and SNU-719 cells). To confirm the regulation of hTERT expression by galectin-3, we determined expression levels of mTERT in galectin-3 knockout (KO) mouse embryonic fibroblasts (MEFs), and observed a decrease in mRNA expression of hTERT (Figure 4A). Remarkably, it was determined that mRNA expression of galectin-3 was also reduced in mTERT KO MEFs (Figure 4B), suggesting that galectin-3 and mTERT regulate each other.

Next, we determined the effect of hTERT on the function of galectin-3 in cell proliferation (Figure 4C). Overexpression of galectin-3 increased the proliferation of SNU-638 cells; however, concomitant 3silencing hTERT reduced this galectin-3-induced cell proliferation. We determined the effect of hTERT on galectin-3-related cellular senescence (Figure 4D and 4E). After overexpression of galectin-3, hTERT expression was increased, whereas p21^cip1^ and p27^kip1^ was decreased. Notably, this decrease in expression induced by galectin-3 was restored by hTERT knockdown (Figure 4D). However, neither galectin-3 nor hTERT affected p53 expression levels (Figure 4D). Overexpression of galectin-3 reduced the amount of SA-β-gal positive SNU-638 cells, which was re-increased by concomitant knockdown of hTERT (Figure 4E). Our results suggest that galectin-3 regulates cell proliferation and cellular senescence in an hTERT-dependent manner.

### hTERT function is regulated by galectin-3 in gastric cancer cells

**Figure 5:**
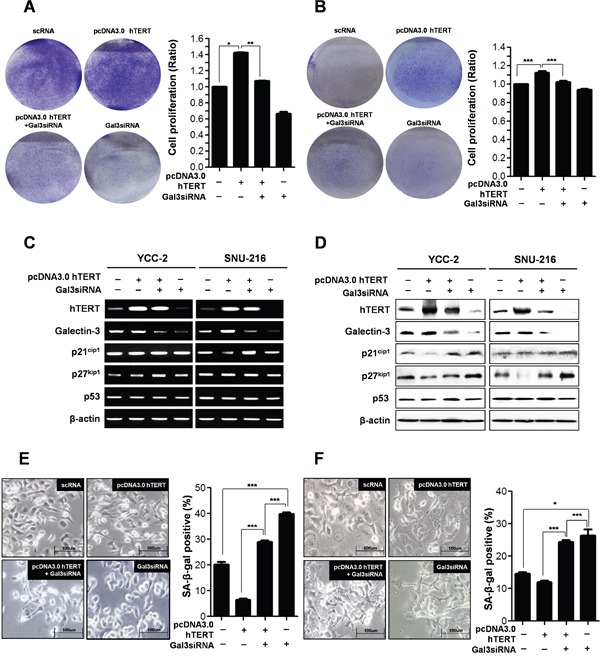
Depletion of galectin-3 after hTERT overexpression on the cell proliferation and cellular senescence of human gastric cancer cells **A.** YCC-2 gastric cancer cells were transfected with scRNA alone, pcDNA3.0-hTERT alone, pcDNA3.0-hTERT and galectin-3 siRNA, and galecitn-3 siRNA alone for 48 hr. Each cell plate was stained with Crystal violet solution and showed by photographs (left panel), and cell proliferation in each cell plate was measured by WST assay and presented by quantitative graphs (right panel). **B.** SNU-216 gastric cancer cells were transfected with scRNA alone, pcDNA3.0-hTERT alone, pcDNA3.0-hTERT and galectin-3 siRNA, and galecitn-3 siRNA alone for 48 hr. Each cell plate was stained with Crystal violet solution and showed by photographs (left panel), and cell proliferation in each cell plate was measured by WST assay and presented by quantitative graphs (right panel). **C.** Detection of mRNA expression levels of hTERT, galectin-3, p21^cip1^, p27^kip1^, and p53 were detected by RT-PCR analysis in both YCC-2 and SNU-216 gastric cancer cells. β-actin was used loading control. **D.** Detection of protein expression levels of hTERT, galectin-3, p21^cip1^, p27^kip1^, and p53 were detected by western blot analysis in both YCC-2 and SNU-216 gastric cancer cells. β-actin was used loading control. Cellular senescence was detected by SA-β-galactosidase assay in **E.** YCC-2 and **F.** SNU-216 gastric cancer cells. The graph shows the percentage of SA-β-galactosidase-positive cells.

Next, we determined whether galectin-3 regulates the function of hTERT on the proliferation of gastric cancer cells (Figure 5A and 5B). Overexpression of hTERT in YCC-2 (Figure 5A) and SNU-216 cells (Figure 5B) increased cell proliferation, whereas this effect was reduced by concomitant knockdown of galectin-3 in both cells. The mRNA expression of levels of p21^cip1^, p27^kip1^, and p53 were not altered after overexpression of hTERT and/or knockdown of galectin-3 (Figure 5C). However, overexpression of hTERT reduced protein expression levels of p21^cip1^ and p27^kip1^, which were re-increased after knockdown of galectin-3 (Figure 5D). Additionally, p53 expression level was not affected (Figure 5D). SA-β-gal positive YCC-2 cells were significantly reduced by overexpression of hTERT, but concomitant knockdown of galectin-3 increased the amount of SA-β-gal positive YCC-2 cells (Figure 5E). This phenomenon was detected in SNU-216 cells, too (Figure 5F).

### Function of hTERT is regulated by galectin-3 in human foreskin fibroblast cells

**Figure 6:**
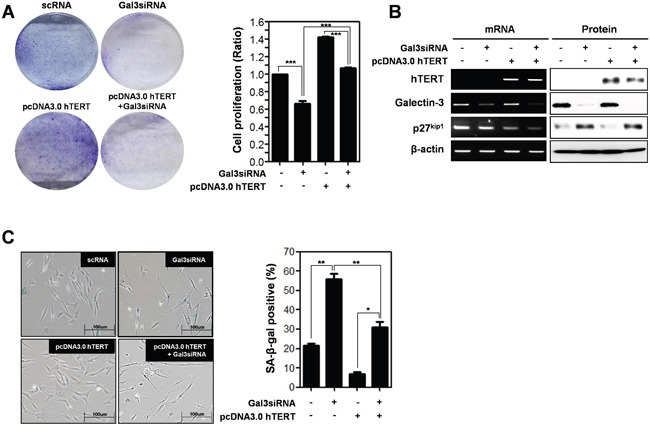
Depletion effect of galectin-3 on the overexpression of hTERT in both the cell proliferation and cellular senescence of human foreskin fibroblasts **A.** Human Foreskin fibroblasts were transfected with scRNA alone, pcDNA3.0-hTERT alone, pcDNA3.0-hTERT and galectin-3 siRNA, and galecitn-3 siRNA alone for 48 hr. Each cell plate was stained with Crystal violet solution and showed by photographs (left panel), and cell proliferation in each cell plate was measured by WST assay and presented by quantitative graphs (right panel). **B.** Detection of mRNA expression levels of hTERT, galectin-3, and p27^kip1^ were detected by RT-PCR analysis and protein expression levels of these were detected by western blot analysis in human Foreskin fibroblasts. β-actin was used loading control. **C.** Cellular senescence was detected by SA-β-galactosidase activity in human Foreskin fibroblasts. The graph shows the percentage of SA-β-galactosidase-positive cells.

To further investigate hTERT-related cellular senescence, we prepared human foreskin fibroblast cells to determine cell proliferation and cellular senescence (Figure 6). Overexpression of hTERT increased the proliferation of fibroblast cells, but concomitant knockdown of galectin-3 reduced the proliferation (Figure 6A). The expression of p27^kip1^ was reduced by overexpression of hTERT, whereas concomitant knockdown of galectin-3 restored the expression levels (Figure 6B). The amount of SA-β-gal positive fibroblast cells was significantly reduced by overexpression of hTERT, whereas concomitant knockdown of galectin-3 re-increased this population (Figure 6C). These data strongly support that galectin-3 regulates both the expression and function of hTERT in cellular senescence.

### Galectin-3 directly interacts with hTERT to regulate its telomeric activity

**Figure 7:**
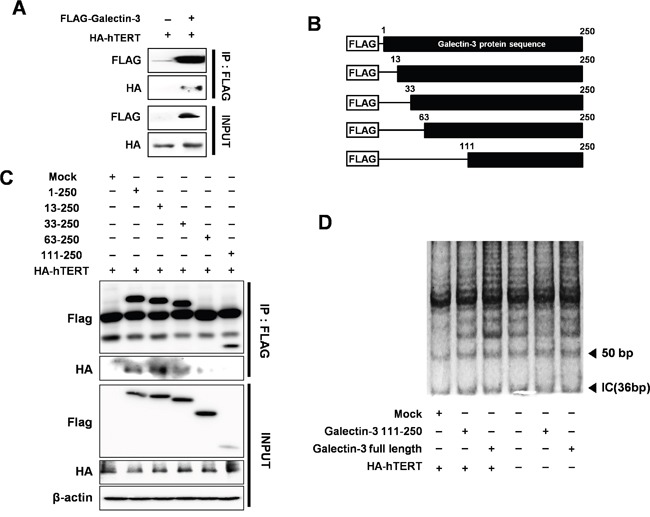
Detection of interaction between hTERT and galectin-3 through the N-terminal domain of galectin-3 and increased telomeric activity after galectin-3 overexpression **A.** Interaction between galectin-3 and hTERT was performed by Immunoprecipitation (IP) assay in HEK293 cells. IP assay was described in “Materials and Methods”. **B.** Schematic model of galectin-3 delayed mutants; Flag-galectin-3 domains (1–250 aa as the full length; 13-250, 33–250, 63–250, and 111–250 aa. **C.** Each deleted mutants of galectin-3 and HA-hTERT were co-transfected in HEK293 cells. IP assay with Flag and HA antibodies to detect the interaction of hTERT with galectin-3 domains were performed **D.** Telomere Repeat Amplification Protocol (TRAP) Assay with galectin-3 binding domain (Full length of galectin-3), galectin-3 wild type, galectin-3 non-binding domain (galectin-3 111–250 AA), and HA-hTERT. TRAP assay was described in “Material and Methods”.

To determine how galectin-3 regulates the function of hTERT, we performed an immunoprecipitation (IP) analysis to detect their interaction (Figure 7A). For this purpose, we prepared plasmids containing domains of galectin-3 (Figure 7B) and performed IP analysis (Figure 7C). Among several domains of galectin-3, overexpression of the domain of 63–250 amino acid or the 111–250 amino acid domain showed no interaction with hTERT, suggesting that the 1–63 amino acid domain could interact with hTERT.

Moreover, we also performed a telomere repeat amplification protocol (TRAP) assay to detect the telomeric activity of hTERT by overexpression of galectin-3 (Figure 7D). Overexpression of full-length galectin-3 induces telomere amplification, whereas overexpression the 111–250 amino acid domains could not induce the same effect, suggesting that galectin-3 enhances the telomeric activity of hTERT.

### Function of hTERT is regulated by galectin-3 in gastric cancer mouse models *in vivo*

**Figure 8:**
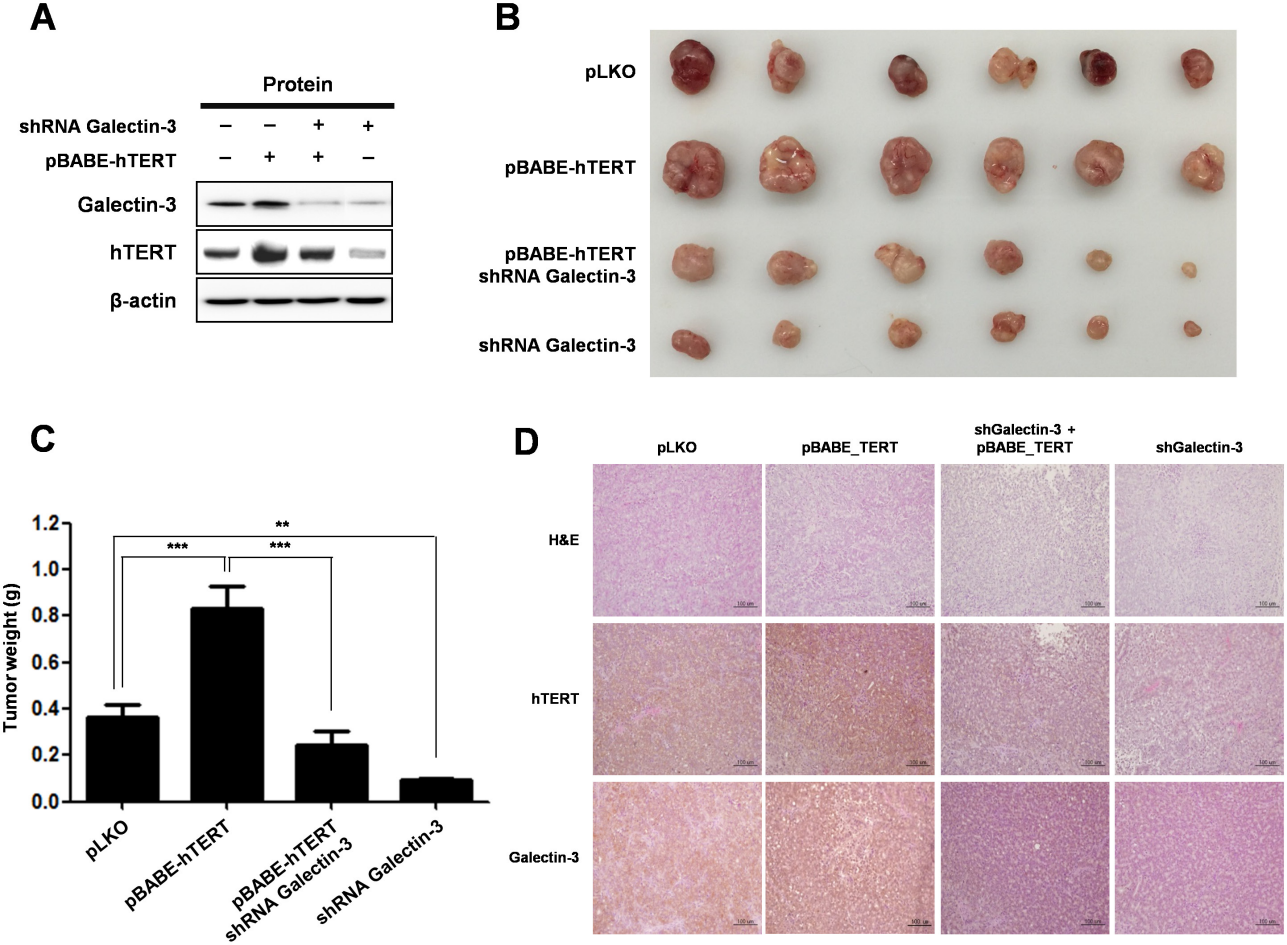
Knockdown of galectin-3 reduces tumor burden in gastric cancer cell xenografted mice, effect that is reversed by overexpression of hTERT **A-D.** Lentivirus expressing galectin-3 specific shRNA or retrovirus overexpressing hTERT was prepared and their employed stable YCC-2 gastric cancer cells were established for *in vivo* study. Lentivirus expressing LacZ shRNA was used as a control and retrovirus overexpressing a vector targeting pBABE-control was used as a control. Mice (n = 5 per group) were inoculated subcutaneously into both flanks with 1 × 10^6^ cells of each YCC-2 cells. **A.** hTERT overexpression and galectin-3 depletion were confirmed by RT-PCR analysis. β-actin was used loading control. **B-C.** Photographs **B.** and quantification of tumor formation **C.** was performed by measuring tumor size and weight 30 days after inoculation. The error bars indicate 95% confidence intervals: *, *P* = 0.001; **, *P* = 0.0015, two-sided *t*-test. All statistical tests were two-sided. **D.** Immunohistochemistry (IHC) analysis was performed to detect the expression level of galectin-3 and hTERT in tumors in *in vivo* mouse models. Method of IHC analysis was described in “Materials and Methods”. Scale bar presents 100 μM.

To analyze the *in vivo* effect of hTERT overexpression and galectin-3-depletion in gastric cancer tumorigenesis, we prepared stable cell lines (Figure 8A) and xenografted them into nude mice (Figure 8B–8D). YCC-2 cells were prepared as follows: pLKO-vector only expressed, pBABE_hTERT - hTERT over-expressed, pBABE_TERT/shRNA_galectin-3 - hTERT overexpressed and galectin-3 depleted, and shRNA_galectin-3 - galectin-3 depleted. The largest tumor burden was detected in hTERT overexpressed YCC-2 cells xenografted mice (Figure 8B and 8C). However, the tumors that were xenografted by overexpressed hTERT and depleted galectin-3 cells were significantly smaller than those prepared by overexpressed hTERT cells, suggesting that the hTERT-promoted gastric tumor growth was regulated by galectin-3. Furthermore, galectin-3 depleted stable cells did not form tumors as well as in pLKO stable cells.

We also examined expression levels of galectin-3 and TERT in tumor tissues from xenografted mice. Overexpression of hTERT in tumors from YCC-2 cells had unchanged the expression level of galectin-3, whereas hTERT expression was significantly decreased in tumors from galectin-3 depleted YCC-2 cells (Figure 8D). Therefore, we confirmed that TERT expression was also significantly regulated by galectin-3 in *in vivo* gastric tumors.

## DISCUSSION

Telomerase is a reverse transcriptase that adds telomeric repeats to the ends of eukaryotic chromosomes. Human telomerase reverse transcriptase (hTERT) is observed in 80–90% of human tumors including gastric cancer, and plays a critical role in the regulation of telomerase activity, which is responsible for endless cell growth [11, 12]. In this study, we confirmed mRNA expression levels of hTERT in both normal and malignant stomach tissues from gastric cancer patients (data analyzed from the GEO dataset in NCBI). There were no differences in mRNA expression levels of hTERT between normal and malignant stomach tissues in six of eight sets of clinical data from gastric cancer patients. However, the survival probability is significantly different between high and low hTERT expression in malignant tissues from gastric cancer patients. The gastric cancer patients with high expression of hTERT had a statistically poorer survival probability than those with low expression of hTERT. It should be noted that high expression of hTERT mRNA in malignant tissues, but not normal tissues, suggesting the regulation of hTERT function has an effect on tumor malignancy.

First, we determined whether expression of hTERT is important in tumor growth, we investigated the effect of hTERT by loss-of function and gain-of function analysis on gastric cancer cell proliferation. Knockdown of hTERT significantly delayed the growth of gastric cancer cells; conversely, overexpression of hTERT increased their growth. Moreover, we determined the effect of hTERT on cellular senescence in gastric cancer cells. Cellular senescence is defined as a quiescent state of proliferative arrest despite preservation of cell viability and maintained metabolic activity [13]. The permanence of senescence growth arrest enforces the senescence response and suppresses the development of cancer [14]. Telomere shortening is a mark of cellular senescence, a type of aging. Functional telomeres prevent DNA repair machineries, cells that rapidly respond and attempt repair, but shortened telomeres lose their function, and cells are forced to cell cycle arrest and cellular senescence. In human cancers, hTERT blocks the shortening of telomeres, thereby preventing genomic instability and enhancing cell cycle progression. In this study, we confirmed that ablation of hTERT significantly enhanced cellular senescence in gastric cancer cells. Notably, ablation of hTERT increased the protein expression of cell cycle inhibitors such as p21^cip1^ and p27^kip1^. However, ablation of hTERT did not effect on the expression of p53. Usually, shortened telomeres elicit a DNA damage response, but do not attempt DNA repair, which in turns activates p53 [15]. In this study, we used both cells; YCC-2 cells are p53 wild-type and SNU-216 cells are p53 hot-spot mutated. However, knockdown or overexpression of hTERT could not change p53 expression, strongly suggested that hTERT regulates cellular senescence in a p53-independent manner in gastric cancer cells. We are able to suggest the non-telomeric activity of hTERT is also important in cellular senescence and gastric tumorigenesis [16]. Previously, we determined the telomerase activity-independent TERT function may contribute to cancer development and aging independently of telomere lengthening [17]. It is also published the non-telomeric activity of TERT [18, 19]. They determined that TERT directly regulates expression of Wnt/β-catenin target genes, including Myc, through physical association. Wnt/β-catenin signaling could regulate the expression level of p21^cip1^ and p27^kip1^ [20]. It is interesting how hTERT regulates the expression level of p21^cip1^ and p27^kip1^, but it is needed to further study.

We explored another pathway which induces cellular senescence, the p16^INK4a^/pRB pathway. We detected a decrease in Rb phosphorylation. However, we could not detect the expression of p16^INK4a^ in gastric cancer cells used in this study. Previously, we demonstrated that galectin-3 interacts with Rb and regulates its phosphorylation [9], and knockdown of galectin-3 decreased the mRNA expression of hTERT [10]. Therefore, we examined whether galectin-3 regulates the expression of hTERT and its function on the regulation of cellular senescence. Knockdown of galectin-3 significantly decreased hTERT expression levels in gastric cancer cells. The expression of mTERT was diminished in galectin-3 KO MEFs. Knockdown of galectin-3 induced-cellular senescence was decreased by overexpression of hTERT in gastric cancer cells. It was determined that the telomeric activity of hTERT was regulated by galectin-3. These results strongly suggested that galectin-3 regulates hTERT expression and telomeric activation.

Interestingly, we detected that the expression of galectin-3 was reduced in mTERT KO MEFs. It suggested that galectin-3 and hTERT modulate each other. As evidence, we determined the direct interaction between hTERT and galectin-3. This direct interaction could regulate the functions of each other, at least on the induction of cellular senescence. It is still unknown how TERT regulates the expression level of galectin-3. However, the expression level of p21^cip1^ and p27^kip1^ and the induction of cellular senescence could be occurred through TERT regulated-galectin-3.

Taken together, we considered that high expression of hTERT leads to poor survival probability in gastric cancer patients and the up-regulated activity of hTERT could be a cause in the development of gastric cancer. Ablation of hTERT significantly induced cellular senescence and inhibited the proliferation of gastric cancer cells. Furthermore, the expression and activity of hTERT on cellular senescence was regulated by galectin-3. Therefore, this study suggests that hTERT could be potent prognostic and therapeutic targets in gastric cancer therapy; further galectin-3 is an important regulator of hTERT expression and telomeric activity in gastric tumorigenesis.

## MATERIALS AND METHODS

### Cell culture and siRNA transfection

The 12 human gastric cancer cell lines (AGS, MKN28, YCC-2, KATOIII, SNU-1, SNU-5, SNU-16, SNU-216, SNU-601, SNU-638, SNU-668, and SNU-719) obtained from the Korea Cell Line Bank were cultured in RPMI 1640 medium supplemented with 5% fetal bovine serum (FBS) and 1% Antibiotics (Invitrogen) and maintained at 37°C in a humidified incubator with a 5% CO_2_ atmosphere as previously described [21–24]. Human galectin-3 and human *TERT* (hTERT) siRNA transfection was performed with Lipofectamine RNAiMAX reagent (Invitrogen) following manufacturer's instructions [25]. The coding strand of galectin-3 siRNA was 5′-AUAUGAAGCACUGGUGAGGUCUAUG-3′ and was purchased from Invitrogen; the coding strand of hTERT siRNA was 5′-UGAUUUCUUGUUGGUGACAUU-3′ and was purchased from Genolution (Seoul, Korea) [26].

### RNA isolation and reverse transcription-polymerase chain reaction (RT-PCR)

Total RNA was extracted from human gastric cancer cells and gastric cancer patient tissues using the TRIzol reagent (Invitrogen) according to the manufacturer's protocol [27]. The RT reaction was carried out using a Reverse Transcription system (Promega), and the PCR was performed using the Ex-taq DNA polymerase (TaKaRa). The primer sequences were as follows: galectin-3: 5′-CAGTGCTCCTGGAGGCTATC-3′ (sense) and 5′-AAGGGGAAGGCTGACTGTCT-3′ (anti-sense); hTERT: 5′-GAACTTGCGGAAGACAGTGG-3′ (sense) and 5′-ATGCGTGAAACCTGTACGCCT-3′ (anti-sense); mTERT; 5′-ATGACCCGCGCTCCTCGTTGC-3′ (sense) and 5′-GACAGCAGAGATGTGGAGCTG-3′ (anti-sense); p21^cip1^: 5′-ATGAAATTCACCCCCTTTCC-3′ (sense) and 5′-CCCTAGGCTGTGCTCACTTC-3′ (anti-sense); p27^kip1^: 5′-AGATGTCAAACGTGCGAGTG-3′ (sense) and 5′-TCTCTGCAGTGCTTCTCCAA-3′ (anti-sense); p53: 5′-GGCCCACTTCACCGTACTAA-3′ (sense) and 5′-GTGGTTTCAAGGCCAGATGT-3′ (anti-sense); β- actin: 5′-AGCCTCGCCTTTGCCGA-3′ (sense) and 5′-CT GGTGCCTGGGGCG-3′ (anti-sense).

### Cell proliferation detection assay

YCC-2, SNU-216, MKN-28, SNU-638, and human fibroblast cells were plated in 96-well culture plates (3 × 10^3^ cells/well). After incubation for 24 h, YCC-2 and SNU-216 cells were transfected with scRNA and hTERT siRNA. MKN-28 and SNU-638 cells were transfected with an empty vector and an hTERT overexpression vector. After transfection for 48 h, the WST-1 solution (Daeil, Korea) was subsequently added to each well. After 1 h of additional incubation, the plate was shaken gently. The absorbance was measured on an ELISA reader at a test wavelength of 450 nm [28, 29].

### Western blot analysis

Western blot analysis was carried out as described previously [30, 31]. Briefly, cells were lysed in RIPA buffer (Biosesang, Seoul, Korea) containing a protease inhibitor cocktail (Sigma), followed by sonication on ice. The cell lysate was centrifuged and the supernatant was collected. Then, 20 μg of protein was subjected to SDS-PAGE and transferred to PVDF transfer membranes (GE healthcare). After being blocked with 5% skim milk for 1 h, the membrane was incubated with a primary antibody dissolved in 5% BSA overnight at 4°C. After that, the membrane was incubated with a secondary antibody for 1 h, followed by detection with an ECL kit (GE healthcare) using LAS 3000. The following antibodies were used: anti-β-actin, anti-Rb, anti-pRb, anti-galectin-3, anti-p21, anti-p27, anti-p53, and anti-TERT (Santa Cruz).

### Immunoprecipitation assays

Cell lysate containing 750 μg of protein was pre-cleared by incubation with 40 μL of protein-A/G linked agarose beads (Santa Cruz) for 1 h at 4°C. After the beads were spun down, the supernatant was incubated with 1 μg of a specific antibody (anti-HA or anti-FLAG, respectively) overnight at 4°C, followed by incubation with 40 μL of protein-A/G agarose beads for 1 h. Mouse IgG (Santa Cruz) was used as the negative control. After the incubation, beads were washed 3 times in RIPA buffer before being dissolved in SDS-PAGE loading buffer. Western blot analysis was performed as described above.

### Infection of galectin-3 shRNA expressing lenti-viral vectors

Galectin-3 shRNA was purchased from Sigma shRNA Bacterial Glycerol Stock. Lentivirus particles were generated using three plasmids: VSVG, RSV-REV, and PMDLg/pPRE, and were co-transfected with galectin-3 shRNA in HEK293FT cells that were transfected as described previously [32].

### Infection of hTERT pBABE expressing retro-viral vectors

pBABE-neo-hTERT was obtained from Addgene [33]. Retrovirus particles were generated using two plasmids, VSVG and GAG/POL, and were co-transfected with pBABE-neo-hTERT in HEK293FT cells that were transfected as described previously.

### TRAP assay

TRAP was performed as previously described [34]. Briefly, cells were lysed with 1 X CHAPS powder (GE Healthcare, MO, USA) containing RNase inhibitor on ice for 30 min. After centrifugation at 13,000 rpm for 10 min, the protein concentration in the supernatant was determined to then incubate at 30°C for 20 min for telomere extension. The extended products were PCR amplified using TS and ACX primers for 33 cycles (denaturation at 94°C for 30 s, annealed at 59°C for 30 s and extended at 72°C for 60 s). NT and TSNT primers were added as an internal control. The PCR products were separated by 12.5% non-denaturing PAGE in 0.5 TBE. Silver staining was carried out as previously described [35]. After electrophoresis, the gels were fixed with a solution containing 0.5% acetic acid and 10% ethanol for 15 min and stained with 0.2% AgNO_2_ for 10 min. The stained gel was quickly washed and developed with 0.1% formaldehyde and 3% NaOH for 20 min.

### Preparation of human gastric cancer xenografted mice

All animal experiments were approved by the Institutional Review Board of Yonsei University College of Medicine and performed at specific pathogen-free facilities under conditions in accordance with the Guidelines for the Care and Use of Laboratory Animals Resources of Yonsei University College of Medicine. The preparation of xenografted mice was performed as described previously [36, 37].

### Statistical analysis

Significant differences between treatment and control groups were determined using the Student's paired *t*-test and ANOVA for multiple samples, if indicated. Differences were considered significant if the *P* value was less than 0.05. Analysis of data was performed using the Prism 5 software.

### Kaplan-meier analysis of relapse-free survival

Kaplan-Meier analysis of survival curve was generated using the online resource http://kmplot.com/analysis and gene set for gastric cancer patients.

## SUPPLEMENTARY MATERIALS FIGURES


