# Supplementary material for: Ablation of human telomerase reverse transcriptase (hTERT) induces cellular senescence in gastric cancer through a galectin-3 dependent mechanism
Source: Oncotarget. 2016 Aug 1;7(35):57117–30. doi: 10.18632/oncotarget.10986 (PMC5302977; doi:10.18632/oncotarget.10986)
Supplement: Supplementary file 1 [file oncotarget-07-57117-s001.pdf]

Ablation of human telomerase reverse transcriptase (hTERT) induces cellular senescence in gastric cancer through a galectin-3 dependent mechanism

SUPPLEMENTARY FIGURES

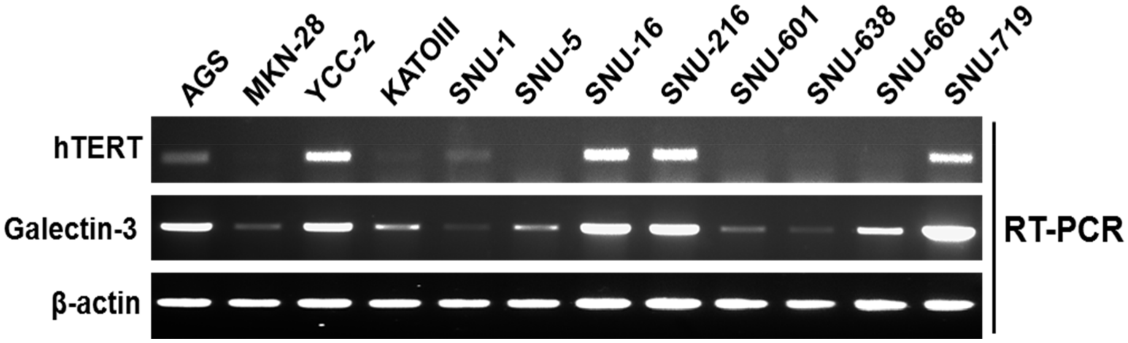

Supplementary Figure S1: mRNA expression levels of *hTERT* and galectin-3 in 12 gastric cancer cell lines. mRNA expression levels of both *hTERT* and galectin-3 were detected by RT-PCR. *β-actin* was used as a normalization loading control.

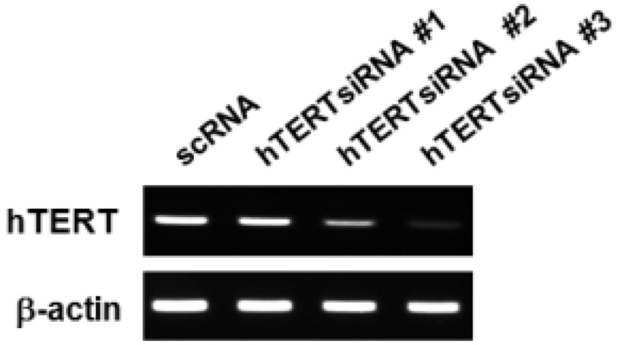

Supplementary Figure S2: Determination of the expression level of *hTERT* by three kinds of h*TERT* specific siRNA in YCC-2 cells. Three kinds of 10 μM *hTERT*-specific siRNA were treated in YCC2 cells for 2 days, the mRNA expression of hTERT was determined by RT-PCR. *β-actin* was used as a normalization loading control.

<Galectin-3 microarray>

Probe lists	Gene symbols	scRNA	Gal3 siRNA	Gal3 siRNA vs scRNA
1555271_a_at	hTERT	60.2	13.8	-2.13
207199_at	hTERT	54.8	43.4	-0.47

Supplementary Figure S3: Silencing galectin-3 with siRNA in gastric cancer cells results in changes in hTERT expression. Levels of hTERT expression and fold changes were confirmed by DNA microarray analysis. DNA microarray results were uploaded on GEO (GSE29630).
